# Predictive value of first-trimester GPR120 levels in gestational diabetes mellitus

**DOI:** 10.3389/fendo.2023.1220472

**Published:** 2023-09-29

**Authors:** Qingwen He, Mengyuan Lin, Zhenhong Wu, Renqiang Yu

**Affiliations:** ^1^ Department of Public Health, Women’s Hospital of Jiangnan University, Wuxi, China; ^2^ Center of Reproductive Medicine, Women’s Hospital of Jiangnan University, Wuxi, China; ^3^ Department of Neonatology, Wuxi Maternity and Child Health Care Hospital, Women’s Hospital of Jiangnan University, Wuxi, China

**Keywords:** gestational diabetes mellitus, biomarker, GPR120, nomogram, LASSO

## Abstract

**Background:**

Early diagnosis of gestational diabetes mellitus (GDM) reduces the risk of unfavorable perinatal and maternal consequences. Currently, there are no recognized biomarkers or clinical prediction models for use in clinical practice to diagnosing GDM during early pregnancy. The purpose of this research is to detect the serum G-protein coupled receptor 120 (GPR120) levels during early pregnancy and construct a model for predicting GDM.

**Methods:**

This prospective cohort study was implemented at the Women’s Hospital of Jiangnan University between November 2019 and November 2022. All clinical indicators were assessed at the Hospital Laboratory. GPR120 expression was measured in white blood cells through quantitative PCR. Thereafter, the least absolute shrinkage and selection operator (LASSO) regression analysis technique was employed for optimizing the selection of the variables, while the multivariate logistic regression technique was implemented for constructing the nomogram model to anticipate the risk of GDM. The calibration curve analysis, area under the receiver operating characteristic curve (AUC) analysis, and the decision curve analysis (DCA) were conducted for assessing the performance of the constructed nomogram.

**Results:**

Herein, we included a total of 250 pregnant women (125 with GDM). The results showed that the GDM group showed significantly higher GPR120 expression levels in their first trimester compared to the normal pregnancy group (p < 0.05). LASSO and multivariate regression analyses were carried out to construct a GDM nomogram during the first trimester. The indicators used in the nomogram included fasting plasma glucose, total cholesterol, lipoproteins, and GPR120 levels. The nomogram exhibited good performance in the training (AUC 0.996, 95% confidence interval [CI] = 0.989-0.999) and validation sets (AUC=0.992) for predicting GDM. The Akaike Information Criterion of the nomogram was 37.961. The nomogram showed a cutoff value of 0.714 (sensitivity = 0.989; specificity = 0.977). The nomogram displayed good calibration and discrimination, while the DCA was conducted for validating the clinical applicability of the nomogram.

**Conclusions:**

The patients in the GDM group showed a high GPR120 expression level during the first trimester. Therefore, GPR120 expression could be used as an effective biomarker for predicting the onset of GDM. The nomogram incorporating GPR120 levels in early pregnancy showed good predictive ability for the onset of GDM.

## 1 Introduction

Gestational diabetes mellitus (GDM), a common gestational disorder, is a growing public health problem worldwide (1). GDM could cause detrimental short- and long-term consequences for the newborn and mother (2–4). In recent years, with improvements in the living standard, changes in diet and lifestyle, and implementation of the “Comprehensive Three Child” policy, there has been an increase in the prevalence of GDM (5). The occurrence of diabetes during the pregnancy period has become an epidemic (4), increasing the health and economic burden in China (6). GDM may not only reflect but also promote the type 2 diabetes mellitus (T2DM) epidemic (7, 8). Women with GDM show a higher probability of developing postpartum T2DM and cardiovascular diseases. Previous studies have shown that early detection of GDM is important for its prevention and treatment (9–12).

Multiple traditional risk factors affect the onset of GDM, such as age, lifestyle, body mass index (BMI) before pregnancy, environmental and psychosocial factors, disorders of lipid metabolism (13, 14), placental hormones (15), fasting plasma glucose (FPG) levels (16), and thyroid functions (17, 18). However, these risk factors have limited diagnostic accuracy. The values of area under the curve (AUC) displayed by the traditional clinical variables was <0.8, while a majority of the models showed a poor agreement between the predicted probability and observed risk (i.e., calibration) (19, 20). The existing predictive model for GDM did not display a considerable or high predictive ability. Therefore, a standard predictive model for the diagnosis of GDM during early pregnancy is necessary (21).

Several researchers have highlighted the correlation between abnormal glucose levels, GDM, and blood lipid metabolism disorders (5, 22). The specific receptor for long-chain fatty acids includes the G-protein-coupled receptor 120 (GPR120), also called the free fatty acid receptor 4 (23). GPR120 is involved in energy metabolism and adipogenesis in adipose tissues and is involved in the onset and progression of several diseases. Our earlier study indicated that the participants in the GDM group exhibited significantly higher GPR120 expression levels compared to the normal healthy controls at 32 and 37 weeks of pregnancy, however, these variations were absent by the second day after delivery (24). Additional lipidomic studies have highlighted the positive correlation between the GPR120 expression levels and total lipid amount in GDM patients (24). Activation of GPR120 reportedly shows a potential therapeutic effect on metabolic syndrome and improves systemic insulin sensitivity in T2DM (25–28). Da et al. noted that GPR120 agonist treatment of the high-fat diet-fed obese mice led to decreased hepatic steatosis, decreased hyperinsulinemia, enhanced glucose tolerance, and increased insulin sensitivity (26). Owing to the similarity between the pathogeneses of GDM and T2DM, GPR120 expression may be correlated with the risk of GDM in the first trimester.

While our previous study has revealed that the expression of GPR120 was significantly higher in the GDM than in the control (24), all these previous studies were based on univariate analyses, and the complicated interactions among multiple male factors were not considered, which may cause biases. Therefore, this study aimed to examine GPR120 levels in patients with GDM in the first trimester and establish an effective predictive model for GDM during the early months of pregnancy.

## 2 Materials and methods

### 2.1 Data collection

**Figure 1:**
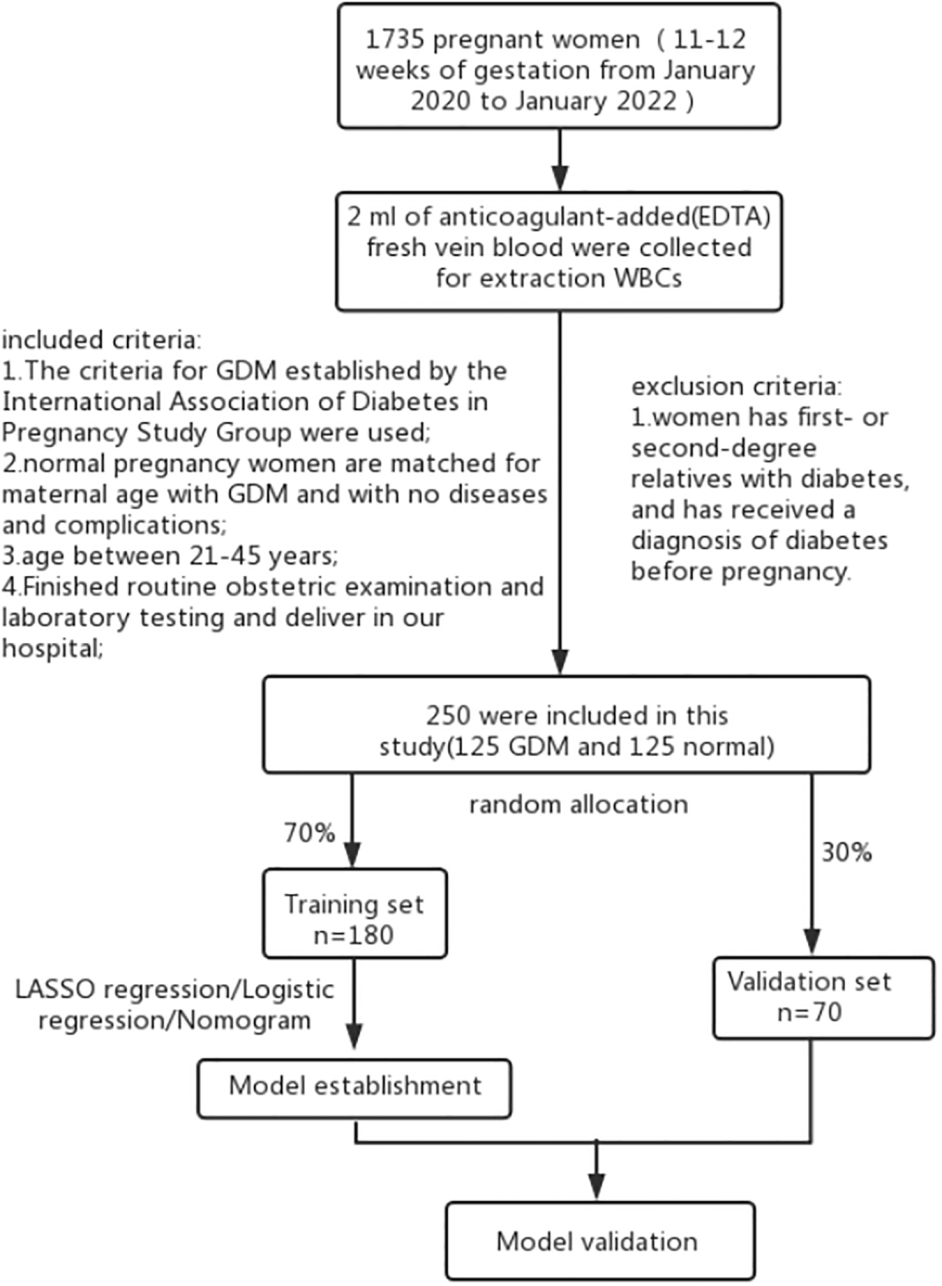
Study flow chart. The nomogram was evaluated based on the AUC-ROC values, calibration curve, C-index, and DCA.

This prospective cohort study recruited 1735 women in the first trimester of pregnancy at Women’s Hospital of Jiangnan University between January 2020 and January 2022. Blood samples were collected from the first-trimester participants. The women at 24-28 weeks of pregnancy were classified into the GDM or control groups depending on the findings of the 75-g oral glucose tolerance test. **Figure 1** presents the inclusion and exclusion criteria used in the study. Herein, 180 pregnant women were enrolled in the training dataset, while 70 women were enrolled in the validation dataset. Thereafter, their laboratory and clinical data, during the 14^th^ –16^th^ gestational week, were collected. The following maternal laboratory and clinical data, which included their systolic blood pressure, age, diastolic blood pressure, gestational week, maternity history, pre-pregnancy BMI, nulliparous, pregnancy BMI, total bilirubin, direct bilirubin, total protein (TP), globin, albumin (ALB), alanine aminotransferase, aspartate aminotransferase, fasting plasma glucose (FPG), creatine kinase, creatinine, uric acid (UA), β2-microglobulin, total cholesterol (TC), low-density lipoprotein (LDL), high-density lipoprotein (HDL), apolipoprotein A1, lipoprotein, apolipoprotein B, *in vitro* fertilization (IVF), and GPR120. Skilled nurses collected the blood samples from the patients, and all blood tests were conducted and management in the laboratory of the Women’s Hospital of Jiangnan University (24). The expression levels of the laboratory factors, except GPR120, were obtained from patient medical records. While the GDM criteria that were defined by the International Association of Diabetes in Pregnancy Study Group were employed in this study (29). The Ethics Committee of the Women’s Hospital of Jiangnan University approved all the experiments conducted in this prospective cohort study (No. 2022-01-1103-15).

### 2.2 Determination of GPR120 mRNA levels in white blood cells

GPR120 mRNA expression levels were determined using white blood cells (WBCs). Firstly, fresh anticoagulant-containing venous blood samples (2 mL) were centrifuged at 2500× g for 10 mins, and the cell-free plasma supernatant layer was removed. Then, red blood cell lysis buffer (10 mL) was gently added to the cell pellet with a pipette, mixed, and gently shaken for 5 mins. This mixture was centrifuged at 2500× g for 5 mins. This pyrolysis step was carried out twice. The cell pellet was rinsed twice with a phosphate-buffered saline solution (3 mL). TRIzol reagent was used for extracting the total RNA content in the WBCs (Tianwei, Beijing, China) following the manufacturer’s recommendations. The Primer Premier 5.0 Software (PREMIER Biosoft International, Palo Alto, CA) was used for designing the GPR120 primers, with the following primer sequences: GPR120: forward 50 -TGG AGC CCC ATC ATC ATC AC-30, reverse 50 - TGC ACA GTG TCA TGT TGT AGA G-3’; The QuantiTect SYBR Green PCR Kit (QIAGEN, Shanghai, China) was utilized for conducting the quantitative polymerase chain reaction (PCR) using the iCycler iQ (Bio-Rad) PCR instrument.

### 2.3 Statistical analysis

The data were statistically analyzed with the use of the R statistical software ver. 4.1.3 (R Statistical Computing Foundation, Vienna, Austria; glmnet, rms, foreign, pROC, regplot, and Nricens packages). The data that conformed to a normal distribution are expressed as mean ± standard deviation, while the nonnormal distributed data are presented as median (interquartile range). Additionally, the categorical data are described as counts and percentages. The summary statistics between the two groups were compared by the Mann–Whitney U test or unpaired Student’s t-tests for continuous data, and chi-square tests for categorical data. The least absolute shrinkage and selection operator (LASSO) regression analysis was conducted for identifying the optimal predictive factors (30). Finally, a nomogram was constructed with the help of the binary logistic regression model with 5-fold cross-validation. The predictive model’s accuracy was determined using the calibration curve (the Hosmer–Lemeshow test was employed for evaluating goodness of fit). Furthermore, the AUC-based receiver operating characteristic (ROC) curves were utilized for evaluating the model’s discriminative ability. Also, the ROC was employed for generating the decision curve analysis (DCA) curves for determining the clinical application and benefit of the nomogram, while the best diagnostic model was chosen depending on the minimal Akaike Information Criterion (AIC). Statistical significance was established at p < 0.05.

## 3 Results

### 3.1 Clinical and laboratory characteristics

This study recruited 125 women with GDM and 125 healthy controls. Among these, the training set included 180 (70%) randomly assigned participants, while the validation set included 70 (30%) randomly assigned participants. **Table 1** presents the basic characteristics and clinical parameters employed in the study cohort. Although the GDM and control groups were matched in terms of age, significant differences were noted between both the groups with regards to their systolic blood pressure, gestational age, pre-pregnancy BMI, pregnancy BMI, and TP, ALB, globin, UA, β2-microglobulin, FPG, TC, HDL, LDL, apolipoprotein B, apolipoprotein A1, lipoprotein, IVF, and GPR120 levels. Participants in the GDM group showed a significantly higher GPR120 expression level compared to the control individuals. The other factors exhibited no statistically significant variation (**Table 1**).

**Table 1:** Comparison clinical and laboratory variables between the two groups.

Variables	GDM( $\bar{\text{x}}$ ± S/ M(IQR)) (N=125)	Control( $\bar{\text{x}}$ ± S/ M(IQR)) (N=125)	Z/t/χ2	*P*
age	31.00(29.00,34.00)	31.00(29.00,34.00)	-1.430	0.153
Gestational weeks (n (%))			1.224	0.542
10	19(15.20)	25(20.00)		
11	83(66.40)	81(64.80)		
12	23(18.40)	19(15.20)		
Systolic blood pressure (mmHg)	117.9±11.44	114.30±10.07	2.642	0.009
Diastolic blood pressure (mmHg)	69.01±9.45	67.82±8.30	1.059	0.290
Maternity history (n (%))			3.120	0.210
0	79(63.20)	91(72.80)		
1	38(30.40)	30(24.00)		
2	8(6.40)	4(3.20)		
Pre-pregnancy BMI(Kg/m2)	22.49(20.42,24.89)	21.05(19.37,22.66)	-4.175	<0.001
Pregnancy BMI (Kg/m2)	24.39(22.15,26.96)	21.71(19.35,23.34)	-7.047	<0.001
TBIL(umol/L)	7.60(6.60,9.55)	8.10(6.70,9.75)	-1.461	0.144
Bilirubin direct(umol/L)	2.12(1.75,2.65)	2.23(1.79,2.62)	-0.397	0.691
TP(g/L)	68.31±4.24	69.79±4.30	-2.741	0.007
ALB(g/L)	37.60(36.00,39.95)	40.70(38.9,43.25)	-6.257	<0.001
Globin(g/L)	30.10(28.00,32.60)	28.90(26.90,30.85)	-3.098	0.002
ALT (mmol/L)	12.70(9.55,17.40)	14.20(10.00,23.35)	-1.314	0.189
AST (mmol/L)	17.80(14.90,22.50)	18.40(16.00,23.85)	-1.639	0.101
CK (mmol/L)	32.7(24.4,45.35)	34.60(26.90,42.70)	-0.66	0.509
UA (mmol/L)	243.9(209.00,299.75)	218.10(185.70,245.95)	-4.466	<0.001
Cr(mmol/L)	45.9(41.40,51.05)	46.80(42.95,50.30)	-0.789	0.43
β2-microglobulin (mg/L)	1.88±0.41	1.65±0.36	4.628	<0.001
FPG(mmol/L)	6.39(6.12,6.95)	4.58(4.35,4.86)	-11.445	<0.001
TC(mmol/L)	5.79(5.30,6.56)	4.34(3.81,4.78)	-11.506	<0.001
HDL(mmol/L)	2.16(1.86,2.37)	1.94(1.71,2.17)	-4.207	<0.001
LDL(mmol/L)	3.46(2.82,4.13)	2.59(2.13,3.01)	-8.189	<0.001
Apolipoprotein A1(g/L)	2.03(1.75,2.37)	1.45(1.22,1.74)	-8.714	<0.001
Apolipoprotein B(g/L)	1.04(0.87,1.27)	0.78(0.67,0.91)	-8.239	<0.001
Lipoprotein(mg/L)	334.20(245.00,368.55)	72.20(38.65,117.95)	-12.398	<0.001
IVF(n (%))			3.879	0.049
Yes	109(87.2)	118(94.4)		
No	16(12.8)	7(5.6)		
GPR120(mmol/L)	4.19(2.25,8.00)	0.98(0.66,1.72)	-10.773	<0.001

### 3.2 Constructing a prediction model based on LASSO and logistic regression analyses in the training dataset

**Figure 2:**
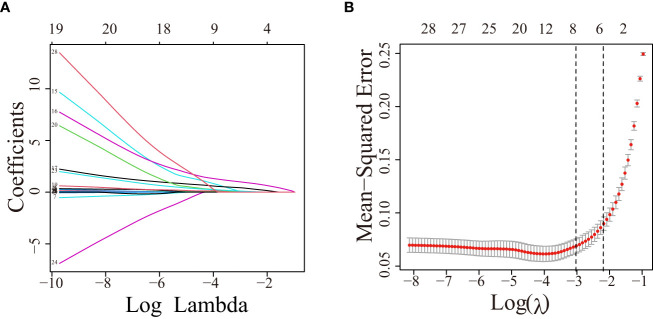
The variable filtering process during LASSO regression. **(A)** Twenty-six variables with non-zero coefficients were chosen by determining the optimal λ. **(B)** After validating the optimal (λ) parameter using the LASSO model, a partial likelihood deviance (binomial deviance) curve was plotted against log (λ), and dotted vertical lines were drawn based on the 1-standard error criteria. LASSO, Least absolute shrinkage and selection operator.

**Figure 3:**
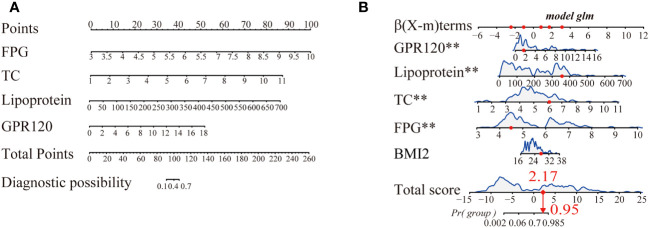
**(A)** A nomogram for predicting gestational diabetes mellitus (GDM). For this purpose, predictor points were determined on the uppermost point scale corresponding to every variable used for the pregnant participants and then added. The numerical value that was projected to the bottom scale highlights the probability of GDM. **(B)** Dynamic nomogram served used as an example. Herein, Participant 1 has been listed as the example (expressed in red). The sum (2.17) of these points is located on the Total score axis, and a line is drawn downward to the probability of developing GDM (95%). **p < 0.01.

**Figure 4:**
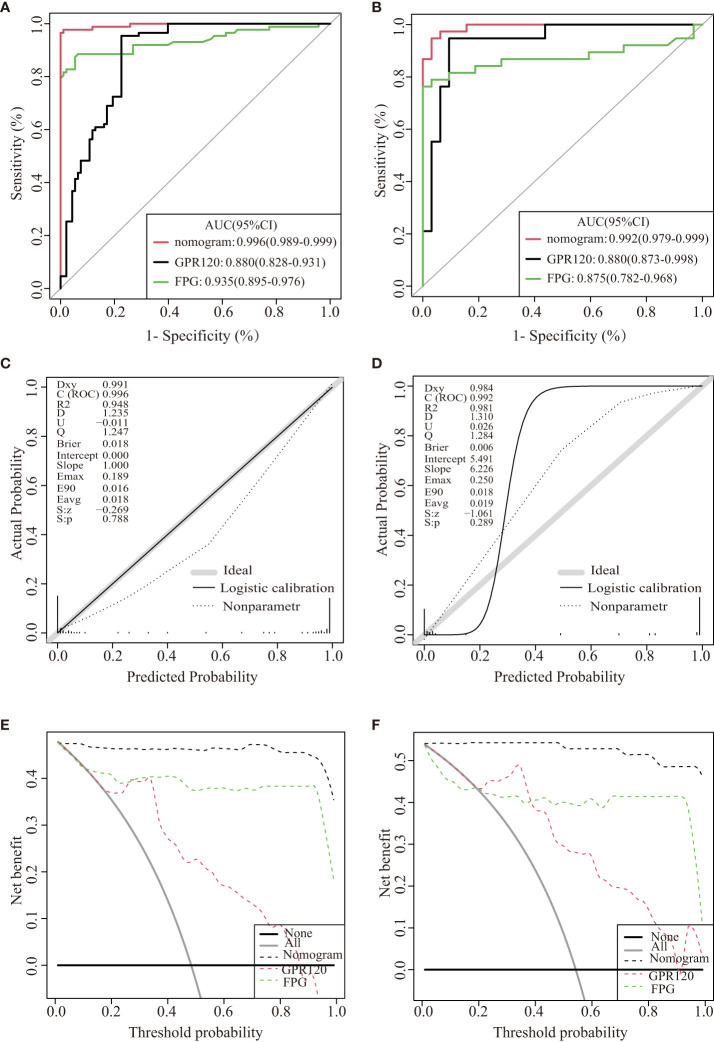
Internal validation of the three models for GDM. ROC curves of the three models for the training dataset **(A)** and validation dataset **(B)**. The calibration curve was derived from the nomogram to predict GDM in the training set **(C)** and validation dataset **(D)**. DCA values were used to predict the performance of the three models in the training **(E)** and validation datasets **(F)**.

Herein, 5 potential predictors with non-zero coefficients were chosen from 26 features for developing the LASSO regression model, including FPG, pregnancy BMI, TC, lipoprotein, and GPR120 levels, which could be used as the GDM risk factors (**Figure 2**). A binomial deviance curve against log (λ) was plotted, where λ indicates the tuning hyperparameter. Furthermore, the solid vertical lines denoted the binomial deviance ± standard error (SE). Also, the 1-SE criteria were employed for drawing the dotted vertical lines at optimal values. The LASSO model used an optimal λ value with the 10-fold cross-validation with 1-SE criterion (**Figure 2B**). The final risk prediction model included FPG, TC, lipoprotein, and GPR120 levels using multivariate logistic regression (**Table 2**). An algorithm that reflected the contribution of these 4 factors to GDM probability (GDMP) was derived from the training cohort data using a logistic regression model: GDMP = 2.504*FPG + 1.528*TC +0.019*Lipoprotein + 0.544*GPR120 - 30.625. **Figure 3** shows the predictive model and its application as a nomogram. For instance, the nomogram model was used for anticipating the probability of a woman with GDM, who showed an FPG level of 4.49 mmol/L, TC levels of 6.11 mmol/L, lipoprotein levels of 356.2 mg/L, and GPR120 levels of 1.68 mmol/L, which was seen to be 95% (**Figure 3B**). In this study, the GPR120 expression level during the first trimester was regarded as an independent risk factor for GDM. Thereafter, the performance of GPR120 as a predictive biomarker for GDM was assessed after developing Model 2 containing only GPR120. As presented in **Figures 4A, B**, Model 2 showed an AUC value of 0.88 (95% confidence interval [CI]: 0.829–0.931) for the training set, while it showed a value of 0.936 (95% CI: 0.873-0.998) for the validation set. Model 2 showed an AIC of 192.73 in the training set. Multivariable logistic regression indicated that the FPG level was significantly and positively related to the higher GDM risk (odds ratio [OR]= 12.236, 95% CI= 2.094–71.494, p = 0.005). FPG is a traditional risk factor for GDM. Therefore, Model 3, which included only FPG levels, was established. The AUC of Model 3 (**Figure 4A**) was 0.935 (95% CI: 0.895–0.976, p < 0.001) for the training set, while it was 0.875 (95% CI: 0.782–0.968) for the validation set. Model 3 showed an AIC of 100.42 for the training set.

**Table 2:** Multivariable logistic regression to predict GDM based on Lasso regression.

Variables	Coefficient	*P* value	Adjusted OR(95%CI)
BMI2	0.191	0.370	1.211(0.797,1.841)
FPG	2.504	0.005	12.236(2.094,71.494)
TC	1.528	0.004	4.609(1.630,13.032)
Lipoprotein	0.019	0.001	1.019(1.008,1.031)
GPR120	0.544	0.001	1.722(1.235,2.402)

### 3.3 Validating the predictive model

The discriminatory abilities of the above three predictive models were determined using the ROC curve. The ROCs of the nomogram were plotted with the data derived from the training and validation datasets. The nomogram showed AUCs of 0.996 (95% CI: 0.989–0.999) and 0.992 (95% CI: 0.9793–0.999) for the training and validation sets, respectively, and the specificity and sensitivity values were 0.977 and 0.989, respectively. The specificity and sensitivity of Model 2(Model 3)for predicting GDM in early pregnancy was 0.954 and 0.774(specificity 0.855 and sensitivity 0.935), respectively. The nomograms showed significantly higher AUCs compared to those displayed by Models 2 and 3 for the training and validation sets.

The nomogram showed an AIC of 37.961. The results implied that the nomogram displayed lower AIC values in comparison to those displayed by the remaining two models displaying the favorable discrimination capability of the nomogram for estimating the likelihood of developing GDM. This predictive model was calibrated by means of the Hosmer–Lemeshow test and calibration plot. The nomogram’s calibration curves exhibited a higher accuracy between the predicted and observed values. The Hosmer–Lemeshow test exhibited a higher consistency between the predicted and actual probabilities (training set, p = 0.788; validation set, p = 0.289) (**Figures 4C, D**). The decision curves for the nomogram in the validation and training sets displayed a relatively good model performance for clinical applications (**Figures 4E, F**). Furthermore, graphical DCA results showed that the nomogram offered a greater net advantage compared to other models over the pertinent threshold range in the entire cohort (**Figures 4E, F**).

## 4 Discussion

In this cohort study, a novel predictive nomogram was constructed that included GPR120 levels and clinical risk factors (such as FPG, TC, and lipoprotein levels). The results indicated that the inclusion of these factors significantly enhanced the nomogram’s ability to detect the onset and progression of GDM in pregnant women in their first trimester. Furthermore, it was noted that the women with GDM showed significantly higher GPR120 expression levels within their first trimester compared to healthy pregnant women. Furthermore, this nomogram displayed a higher level of discrimination and exhibited an AUC of 0.996. Thus, clinicians can use this prediction model to identify the patients showing a high risk of GDM, thus developing effective and targeted treatment strategies.

GDM is a common, comprehensive, obstetric, and gynecological disease syndrome that is related to abnormal lipid and glucose metabolism during pregnancy. Although GDM presents a significant threat to maternal and fetal safety during pregnancy (31), very less information regarding its pathogenesis is available. Our data showed that some women diagnosed with GDM exhibited abnormal glucose and blood lipid metabolism during the first trimester (**Table 1**). Wang et al. found that lipid metabolism disorders noted in the early months of pregnancy were associated with the risk of GDM. Immanuel and Simmons reported that many women with GDM (15–70%) present signs of hyperglycemia before 24 weeks of gestation (5, 32), which was similar to the results presented in this study. Currently, early clinical treatment generally focuses on regulating the patients’ diet and exercise (33, 34) and implementing blood glucose management plans in the first trimester, which are important for both fetal and maternal health (35). However, the GDM diagnosis is generally carried out in the 24^th^–28^th^ weeks of pregnancy, which presents a limited time for intervention. Thus, an early GDM prediction model needed to be developed for improving the prevention, treatment, and prognosis of GDM and decreasing the economic burden (36).

This prospective cohort study recruited 250 patients for constructing a nomogram based on multiple variables that were screened by means of the LASSO regression analysis. The traditional biochemical indicators of GDM exhibit strong collinearity. LASSO regression, which is better than univariate analysis, helps in addressing the issue of multicollinearity among the variables. **Figure 2** illustrates the LASSO penalty selection process. A majority of the earlier studies used statistical techniques that combined univariate analysis and multivariate logistic regression methods for analyzing the data (36, 37). The findings in this report indicated that in comparison to the multivariate logistic regression analysis, a combination of LASSO regression and multivariate logistic regression analyses yields a better AUC. Herein, multivariate logistic regression analyses implied that the TC, FPG, lipoprotein, and GPR120 levels could be used as independent predictive factors for GDM. Earlier studies showed that the FPG and lipoprotein levels were independent risk factors for GDM, which were further validated by the findings noted in this study (10, 11, 38). However, several studies in the past have conducted univariate logistic regression analysis for identifying GDM-related risk factors (39, 40). This may be due to an indirect correlation between exposure and outcome among the research variables included in the model, which makes TC insignificant in the multivariate analysis. This contradictory event demonstrates the disparity between the statistical methodologies as well as the prospective advantages of the multivariate analysis. The findings of the univariate regression analyses indicated the significance of a single factor based on the presumption that this factor operates independently without taking into consideration its interaction with other relevant factors. However, due to the strong interactions between various GDM-related factors, the findings of the univariate analysis could not present a subjective conclusion. A multivariate analysis assists in overcoming these limitations.

GPR120 is involved in the lipid and glucose metabolism processes, where medium-to-long-chain fatty acids serve as ligands (41). Since GDM shows a similar pathology as T2DM, GDM can be regarded as an early T2DM stage (42). GPR120 protects against obesity and T2DM (25–27), however, its actual role in GDM is unclear. However, several hypotheses have been proposed. Fasting plasma glucose (FPG) is diagnosis maker for diabetes. Meanwhile, the main role of GPR120 is to elicit free fatty acids regulation on metabolism homeostasis and GPR120 agonism correlates with prevention of the occurrence and development of metabolic disorders such as obesity and diabetes. Thus, the disorder of GPR120 expression may cause the level of FPG raised. In this study, we demonstrated that GPR120 levels increased the risk of developing GDM. This phenomenon is linked to the upregulated GPR120 expression levels, which protect individuals from various lipid disorders. Therefore, it was speculated that the GPR120 agonists could exhibit a therapeutic value among GDM individuals. However, the mechanism used by GPR120 to regulate lipid metabolism is not defined and needs to be further investigated.

We constructed a nomogram for GDM, which, for the first time, demonstrated that GPR120 expression levels during the first trimester could be utilized for predicting the development of GDM. This nomogram showed a considerable degree of discrimination (AUC = 0.996) and calibration (p = 0.788). Tong et al. reported that FPG could serve as an independent risk factor for GDM during the initial trimester and could be employed as a screening tool for determining risky GDM-related pregnancies and predicting adverse pregnancy outcomes. The findings noted in this study suggested that the developed nomogram showed a better predictive ability compared to the two other models in all cohorts. Therefore, GPR120 was selected to enhance the model’s ability to identify the onset of GDM during the first trimester. Different first-trimester-related GDM nomograms were proposed in the past. However, a majority of GDM risk prediction models that have been established earlier are based on the primary characteristics of pregnant women, like pre-pregnancy BMI or age, and do not include GPR120 levels. Most studies on this topic are retrospective, which restricts the clinical significance of all the results. The previously established nomograms have limited diagnostic accuracy (11, 43, 44), and the AUC of these models is less than 0.8 (36, 45), which is lower than that of our model. Furthermore, the results of the DCA curve showed that the constructed nomogram displayed a positive effect, which validated the better clinical value of this model compared to other models.

Despite the advantages presented in this study, it shows a few limitations. This single-center study had a limited sample size, where the population showed a restricted ethnicity. Furthermore, the mechanism used by GPR120 for GDM regulation is not known. Thus, in the future, multicenter studies with large sample sizes should be conducted for verifying the results noted in this study. Furthermore, the specific mechanism responsible for the interaction between GPR120 and GDM requires further investigation.

## 5 Conclusions

To conclude, patients with GDM showed high GPR120 transcriptional levels during their early trimester. The novel nomogram that was constructed in this study included the GPR 120 levels within the first 3 months of pregnancy, and it displayed good predictive and discrimination values.

## Data availability statement

The original contributions presented in the study are included in the article/supplementary material. Further inquiries can be directed to the corresponding authors.

## Ethics statement

The studies involving humans were approved by Institutional Ethics Committee of the Wuxi Maternity and Child Health Care Hospital. The studies were conducted in accordance with the local legislation and institutional requirements. The participants provided their written informed consent to participate in this study.

## Author contributions

QH and ML are responsible for the collection of data and writing of the original manuscript. RY and are ZW responsible for the concept development, revision, review of the manuscript. RY is responsible for funding acquisition. All authors contributed to the article and approved the submitted version.
